# Resistance profile to antimicrobials agents in methicillin-resistant *Staphylococcus aureus* isolated from hospitals in South Brazil between 2014-2019

**DOI:** 10.1590/0037-8682-0431-2020

**Published:** 2020-11-06

**Authors:** Adriana Medianeira Rossato, Muriel Primon-Barros, Lisiane da Luz Rocha, Keli Cristine Reiter, Cícero Armídio Gomes Dias, Pedro Alves d’Azevedo

**Affiliations:** 1 Universidade Federal de Ciências da Saúde de Porto Alegre, Programa de Pós-graduação em Ciências da Saúde, Porto Alegre, RS, Brasil.

**Keywords:** Methicillin-resistant *Staphylococcus aureus*, Healthcare-associated infections, Antimicrobial susceptibility, Resistance genes

## Abstract

**INTRODUCTION:**

Methicillin-resistant *Staphylococcus aureus* (MRSA) is a common pathogen causing healthcare-associated infections. Owing to the restricted use of beta-lactams in MRSA infections, non-beta-lactam antimicrobials are required for treatment. However, MRSA can develop resistance mechanisms to non-beta-lactam antimicrobials, which reduces viable treatment options. Here, we evaluated the antimicrobial susceptibility and resistance genes of MRSA isolated from hospitalized patients in South Brazil.

**METHODS:**

The antimicrobial susceptibilities of hospital MRSA (217) isolates were determined by disk diffusion or microdilution methods. Additionally, the presence of 14 resistance genes and SCC*mec* typing was performed by PCR.

**RESULTS:**

Among the antimicrobials tested, we observed high erythromycin (74.2%), ciprofloxacin (64.5%), and clindamycin (46.1%) resistance rates and complete susceptibility to linezolid and vancomycin. Seventeen different patterns of MRSA antimicrobial resistance were observed, of which 42.9% represented multidrug resistance. Among erythromycin-resistant MRSA, 53.4%, 45.3%, 37.9%, 13.0%, and 6.8% carried *ermA*, *msrA*, *msrB*, *ermC*, and *ermB* genes, respectively. Among clindamycin-resistant MRSA, 83%, 17%, 10%, 4%, and 2% carried *ermA*, *ermC*, *ermB*, *linA*, and *linB* genes, respectively. Among gentamicin-resistant MRSA, 96.8%, 83.9%, and 9.7% carried *aac(6')/aph(2'')*, *aph(3’)-IIIa*, and *ant(4’)-Ia* genes, respectively. Among tetracycline-resistant MRSA, 6.5% and 93.5% carried *tetK* and *tetM* genes, respectively. Lastly, among trimethoprim/sulfamethoxazole-resistant MRSA, 13.3% and 100% carried *dfrA* and *dfrG* genes, respectively. The SCC*mec* type IV isolates were detected more frequently, whereas the SCC*mec* type III isolates exhibited higher multidrug resistance.

**CONCLUSIONS:**

The study data provides information regarding the MRSA resistance profile in South Brazil that is associated with the clinical conditions of patients and can contribute to clinical decision-making.

## INTRODUCTION

Antimicrobial resistance poses a significant challenge to modern medicine as well as to the possibility of effective treatment of infectious diseases^1^. Methicillin-resistant *Staphylococcus aureus* (MRSA) is one of the most frequent causes of community- and healthcare-associated infections (CA-MRSA and HA-MRSA, respectively). A major concern remains owing to higher morbidity and mortality when compared with infections caused by methicillin-susceptible strains (MSSA), along with increased hospitalization and health care costs^2^. MRSA strains pose a threat to public health owing to their potential for genetic adaptation and remarkable ability to acquire resistance to multiple antimicrobials, along with the implications for the treatment of this pathogen^3^ ^,^ ^4^.

Methicillin resistance is mediated by the acquisition of genes (*mecA* or *mecC*) found in the mobile genetic element called staphylococcal cassette chromosome *mec* (SCC*mec*), which encodes an altered penicillin-binding protein (PBP2a or PBP2’) that confers low affinity for most beta-lactams^5^ ^,^ ^6^. The SCC*mec* elements are classified into thirteen different types (SCC*mec* I-XIII) based on structural organization and genetic content^2^. CA-MRSA strains generally harbor SCC*mec* type IV or V, and are susceptible to non-beta-lactam antimicrobials. HA-MRSA strains commonly harbor SCC*mec* types I, II, or III, which contain genes that confer resistance to non-beta-lactam antimicrobials^7^.

Owing to the restricted use of beta-lactams for treating infections caused by MRSA, non-beta-lactam antimicrobials, such as aminoglycosides, fluoroquinolones, folate inhibitors, glycopeptides, lincosamides, lipopeptide, macrolides, oxazolidinones, and tetracyclines, are required for the treatment of staphylococcal infections. However, these therapeutic options are reduced when MRSA isolates develop resistance mechanisms to survive in conditions with high concentrations of these antimicrobials^4^ ^,^ ^8^ ^,^ ^9^.

Resistance is associated with different molecular mechanisms, as follows: 1) inactivation of antimicrobials by enzymes, such as inactivation of aminoglycosides by aminoglycoside-modifying enzymes (AMEs) (encoded by *aac(6')/aph(2'')*, *aph(3’)-IIIa,* and *ant(4’)-Ia* genes)^10^, trimethoprim by variants of dihydrofolate reductases (DHFRs) (*dfrA* and *dfrG*)^11^, or lincosamide by lincosamide nucleotidyltransferases (*linA* and *linB*)^12^ ^,^ ^13^; 2) alterations in ribosomal binding site (*ermA*, *ermB,* and *ermC*), which confers resistance to macrolides and lincosamides^9^ ^,^ ^12^; 3) active efflux pumps, such as those encoded by *msrA*, *msrB,* and *tetK* ^12^, which impart resistance to macrolides, type B streptogramins, and tetracycline, respectively^14^; and 4) ribosomal protection (*tetM*), that confers resistance to tetracycline^14^. These mechanisms limit the therapeutic options available for the treatment and control of MRSA infections.

The latest data from the Centers for Disease Control and Prevention (CDC) show more than ten thousand deaths caused by MRSA, with high healthcare costs, in the US^15^. In Latin America, the resources for monitoring the epidemiology of MRSA remain limited. Additionally, the true nature and extent of MRSA infections are inadequately known; this indicates that local data collection should be coordinated with effective interventions for making clinical decisions for the control of staphylococcal infections^16^. Considering the importance of global surveillance studies on resistance profiles, along with the current challenges related to the treatment of MRSA infections, this study aimed to evaluate antimicrobial susceptibility and identify the resistance genes in MRSA obtained from hospitals in South Brazil.

## METHODS

### Study design and clinical strains

This cross-sectional observational study was conducted using 217 MRSA isolates obtained between January 2014 and January 2019 (40 in 2014, 49 in 2015, 75 in 2016, 29 in 2017, and 24 in 2018) from hospitals in Porto Alegre in South Brazil. The study was registered under the Institutional Ethics Committee number 2.770.338. The strains were isolated from respiratory tract (75; 34.6%), blood (55; 25.3%), skin and soft tissue (42; 19.4%), bone and connective tissue (24; 11.1%), and sterile cavity liquid (12; 5.5%) samples, and from medical devices (9; 4.1%). The isolates were cryopreserved and stored at -20 °C until testing.

### Identification of *S. aureus*

The isolates were identified as *S. aureus* using conventional microbiological methods, such as evaluation of colony morphology on sheep blood agar, Gram staining, catalase activity, production of coagulase, and growth on mannitol salt agar. Methicillin resistance was confirmed by the cefoxitin disk diffusion method and polymerase chain reaction (PCR) for the detection of *mec*A gene according to Clinical and Laboratory Standard Institute (CLSI) guidelines, 2019^17^.

### Antimicrobial susceptibility tests

The susceptibility of isolates to ciprofloxacin (5 μg), clindamycin (2 μg), erythromycin (15 μg), gentamycin (10 μg), linezolid (30 μg), tetracycline (30 μg), and trimethoprim/sulfamethoxazole (1.25 μg/23.75 μg) was determined by the disk diffusion method on Mueller-Hinton agar (Oxoid, Basingstoke, England).

Clindamycin susceptibility was determined using a disk approximation test with erythromycin and clindamycin (D-test). The following resistant phenotypes were identified in the D-test: inducible phenotype (iMLS_B_), when resistant to erythromycin and susceptible to clindamycin with formation of a D-shaped zone, constitutive resistance phenotype (cMLS_B_) when resistant to both erythromycin and clindamycin, and MS phenotype when resistant to erythromycin and susceptible to clindamycin without formation of a D-shaped zone^18^. 

The minimal inhibitory concentration (MIC) of vancomycin was determined using the microdilution method in Mueller-Hinton broth (Oxoid, Basingstoke, England). The results of antimicrobial susceptibility were interpreted according to the CLSI guidelines^17^. The strains obtained from the American Type Culture Collection (ATTC), *S. aureus* ATCC 25923 and *S. aureus* ATCC 29213, were used as controls.

### Detection of antimicrobial resistance genes

The detection of genes related to antimicrobial resistance, including (*aac(6')/aph(2'')*, *ant(4’)-Ia*, *aph(3’)-IIIa*, *dfrA*, *dfrG*, *ermA*, *ermB*, *ermC*, *linA*, *linB*, *msrA*, *msrB*, *tetK*, and *tetM*), in MRSA was confirmed by conventional PCR, as previously described, with certain modifications (Table 1)^10^ ^,^ ^19^ ^-^ ^23^.

**TABLE 1:** Primer sequences and amplification conditions used to detect resistance genes.

Target gene	Primer sequence (5’-3’)	Amplicon (bp)	Amplification conditions	Ref.
*aac(6')/aph(2'')*	F: CAG AGC CTT GGG AAG ATG AAG	348	Pre cycle: 94 °C -3 min	11
	R: CCT CGT GTA ATT CAT GTT CTG GC			
*ant(4’)-Ia*	F: CAA ACT GCT AAA TCG GTA GAA GCC	294	35 cycles: 94 °C - 40 s,	
	R: GGA AAG TTG ACC AGA CAT TAC GAA		55 °C - 40 s, 72 °C - 40 s	
*aph(3’)-IIIa*	F: GGC TAA AAT GAG AAT ATC ACC GG	523	Last cycle: 72 °C - 2 min	
	R: CTT TAA AAA ATC ATA CAG CTC GCG			
*dfrA*	F: CAC TTG TAA TGG CAC GGA AA	270	Pre cycle: 94 °C - 4 min	19
	R: CGA ATG TGT ATG GTG GAA AG		30 cycles: 94 °C - 1 min,	
			52 °C - 30 s, 72 °C - 1 min	
*dfrG*	F: TGC TGC GAT GGA TAA GAA	405	Last cycle: 72 °C - 4 min	
	R: TGG GCA AAT ACC TCA TTC C			
*ermA*	F: TCT AAA AAG CAT GTA AAA GAA	645	Pre cycle: 93 °C - 3 min	20
	R: CTT CGA TAG TTT ATT AAT ATT AGT		35 cycles: 93 °C - 1 min,	
			52 °C - 1 min, 72 °C - 1 min	
*ermB*	F: GAA AAG GTA CTC AAC CAA ATA	639	Last cycle: 72 °C - 5 min	
	R: AGT AAC GGT ACT TAA ATT GTT TAC			
*ermC*	F: TCA AAA CAT AAT ATA GAT AAA	642	Pre cycle: 93 °C - 3 min	20
	R: GCT AAT ATT GTT TAA ATC GTC AAT		35 cycles: 93 °C - 1 min,	
			53 °C - 1 min, 72 °C - 1 min	
			Last cycle: 72 °C - 5 min	
*linA*	F: GTA TTA ACT GGA AAA CAG CAA AG	323	Pre cycle: 94 °C - 5 min	21
	R: GAG CTT CTT TTG AAA TAC ATG G		35 cycles: 94 °C - 45 s,	
*linB*	F: CCT ACC TAT TGT TTG TGG AA	925	48 °C 45 s, 72 °C - 1 min	
	R: ATA ACG TTA CTC TCC TAT TC		Last cycle: 72 °C - 5 min	
*msrA*	F: GGC ACA ATA AGA GTG TTT AAA GG	940	Pre cycle: 94 °C - 5 min	22
	R: AAG TTA TAT CAT GAA TAG ATT GTC CTG TT		25 cycles: 94 °C - 1 min,	
*msrB*	F: TAT GAT ATC CAT AAT AAT TAT CCA ATC	595	50 °C 1 min, 72 °C - 1 min	
	R: AAG TTA TAT CAT GAA TAG ATT GTC CTG TT		Last cycle: 72 °C - 10 min	
*tetK*	F: CAG CAG ATC CTA CTC CTT	168	Pre cycle: 93 °C - 5 min	21
	R: TCG ATA GGA ACA GCA GTA		35 cycles: 93 °C - 1 min,	
			54 °C 1 min, 72 °C - 1 min	
			Last cycle: 72 °C - 10 min	
*tetM*	F: GTG GAC AAA GGT ACA ACG AG	405	Pre cycle: 93 °C - 5 min	23
	R: CGG TAA AGT TCG TCA CAC AC		35 cycles: 93 °C -1 min,	
			52 °C 1 min, 72 °C - 1 min	
			Last cycle: 72 °C - 10 min	

***aac(6')/aph(2''):*** gene that encodes aminoglycoside-6'-N-acetyltransferase/2''-O-phosphoryltransferase; ***ant(4’)-Ia:*** gene that encodes aminoglycoside-4'-O-phosphoryltransferase I;***aph(3’)-IIIa:*** gene that encodes aminoglycoside-3'-O-phosphoryltransferase III;***dfrA*:** gene that encodes dihydrofolate reductase A; ***dfrG:*** gene that encodes dihydrofolate reductase B; ***ermA:*** gene that encodes erythromycin ribosomal methylase A; ***ermB:*** gene that encodes erythromycin ribosomal methylase B; ***ermC:*** gene that encodes erythromycin ribosomal methylase C; ***linA:*** gene that encodes lincosamide nucleotidyltransferases A; ***linB:*** gene that encodes lincosamide nucleotidyltransferases B; ***msrA:*** gene that encodes macrolides streptogramins resistance A; ***msrB:*** gene that encodes macrolides streptogramins resistance B; ***tetK:*** tetracycline resistance protein K; ***tetM:*** tetracycline resistance protein M.

Bacterial deoxyribonucleic acid was extracted by using Chelex®100 (Bio-Rad, Richmond, CA, USA) and Proteinase K (Sigma-Aldrich, Poole, UK). The PCR reaction contained 0.2 mM of each deoxyribonucleotide triphosphate (10 mM), 2 mM of MgCl_2_ (50 mM), 1X PCR buffer (10 X), 0.5 μM of forward/reverse primers (10 μM), 1.5 U of Taq DNA polymerase (5 U/μL), and 1 μL of DNA template in a total volume of 25 μL. Amplifications were performed using a LifePro Thermal Cycler (Hangzhou Bioer Technology Co. Ltd., Hangzhou, China). The PCR amplicons were separated by electrophoresis in a 2.0% agarose gel (Sigma-Aldrich, USA) and stained with 0.1% ethidium bromide (0.4 μg/mL). 

The PCR-positive controls, *S. aureus* JCSC 4469 (*aac(6’)*/*aph(2”)*), *S. aureus* N315 (*ant(4’)-Ia*), *S. aureus* JCSC 4488 (*aph(3’)-IIIa* and *dfrG*), *S. aureus* WIS (*dfrA*), *S. aureus* NCTC 10442 (*ermA*), *S. aureus* HDE 288 (*ermB*), *S. aureus* JCSC 4474 (*ermC*), *S. aureus* JCSC 6082 (*linA), S. aureus* JCSC 2172 (*linB*), *S. aureus* NCTC 8325 (*msrA* and *msrB*), *S. aureus* 85/2082 (*tetK*), and *S. aureus* JCSC 6943 (*tetM*) were included. A tube containing all components of the PCR mixture, except the template DNA, was used as the negative control. 

### SCC*mec* typing 

The SCC*mec* types I-X were identified by multiplex-PCR, as previously described^24^. The *S. aureus* strains NCTC 10442, N315, 85/2082, JCSC 4474, WIS, HDE 288, JCSC 6082, JCSC 6943, and JCSC 6945 were used as the positive controls for the SCC*mec* types I, II, III, IV, V, VI, VII, IX, and X, respectively. The PCR mixture components without the DNA template were used as negative control.

### Statistical analysis

Sta­tistical analysis was performed using SPSS version 20.0 software (SPSS, Chicago, IL, USA). Chi-square test or Fisher’s exact test was performed to analyze the results. *p* value < 0.05 was considered statistically significant.

## RESULTS

### Antimicrobial susceptibility

**FIGURE 1:**
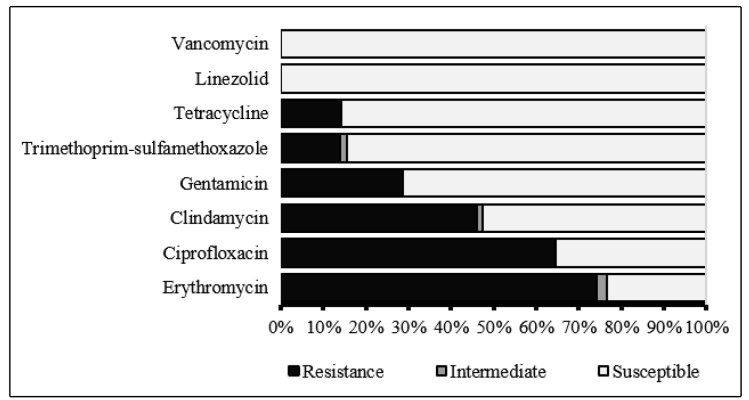
Antimicrobial susceptibility of the methicillin-resistant *Staphylococcus aureus* isolates.

In the antimicrobial susceptibility tests of the MRSA isolates, the highest resistance rates were observed for erythromycin (74.2%; 161/217), ciprofloxacin (64.5%; 140/217), and clindamycin (46.1%; 100/217). Furthermore, 2.3% (5/217) of the isolates exhibited intermediate resistance to erythromycin and 1.4% (3/217) to clindamycin. The overall prevalence of iMLS_B_, cMLS_B_, and MS_B_ phenotypes was 7.4% (16/217), 46.1% (100/217), and 26.3% (57/217), respectively. Conversely, lower resistance rates were observed against gentamicin (28.6%; 62/217), tetracycline (14.3%; 31/217), and trimethoprim-sulfamethoxazole (13.8%; 30/217). Additionally, 1.8% (4/217) of the isolates exhibited intermediate resistance to trimethoprim-sulfamethoxazole. All isolates were susceptible to linezolid and vancomycin, with MIC values to vancomycin of 0.25 μg/mL (41.0%; 89/217), 0.5 μg/mL (26.3%; 57/217), 0.75 μg/mL (16.6%; 36/217), 1 μg/mL (13.4%; 29/217), and 1.5 μg/mL (2.8%; 6/217) (Figure 1).

**FIGURE 2:**
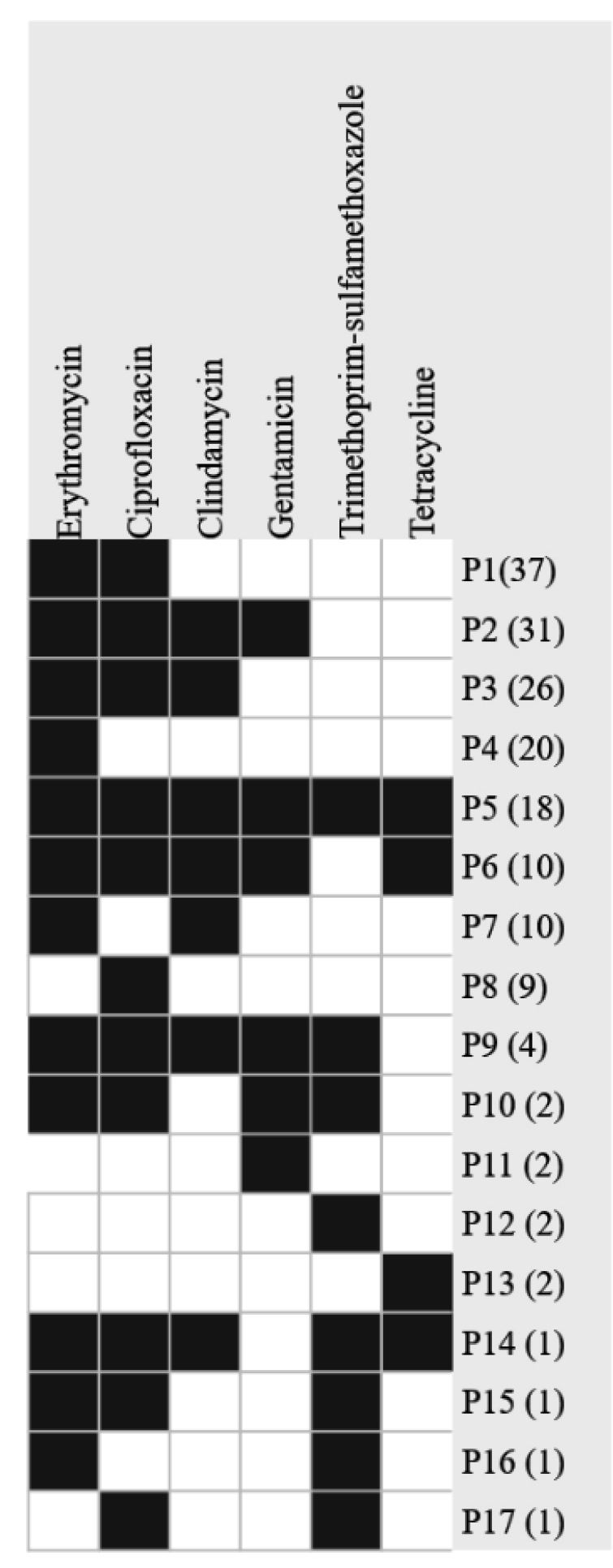
Heat map of antimicrobial resistance patterns among 217 methicillin-resistant *Staphylococcus aureus* isolates.

Among the 177 out of the 217 MRSA isolates that exhibited resistance to non-beta-lactam antimicrobials, we observed 17 distinct patterns (P) of antimicrobial resistance (Figure 2), of which 12 were grouped and 5 were singular patterns. The dominant resistance pattern (P1)-erythromycin and ciprofloxacin resistance-was observed in 37 isolates. The fifth pattern of antimicrobial resistance (P5) was identified in 18 isolates that were resistant to six antimicrobials. Furthermore, resistance patterns to five (P6) and four (P2) antimicrobials were observed in 10 and 31 isolates, respectively (Figure 2).

Upon analyzing the prevalence of MRSA resistance among isolates collected in different years, we observed that 93 isolates (42.9%) exhibited multidrug resistance, i.e., they were resistant to three or more classes of antimicrobial agents, excluding isolates with intermediate resistance. Among them, 32 (34.4%) exhibited resistance against at least three different classes of antimicrobials, 28 (30.1%) against four classes, 15 (16.1%) against five classes, and 18 (19.4%) against six classes.

### Detection of antimicrobial resistance genes

The detection of the resistance genes showed that, among erythromycin-resistant MRSA representing the macrolides class, the most frequently encountered gene was *ermA* (86; 53.4%), followed by *msrA* (73; 45.3%), *msrB* (61; 37.9%), *ermC* (21; 13.0%), and *ermB* (11; 6.8%); 3 (1.9%) isolates tested negative for these genes. In the lincosamides class, among 100 clindamycin-resistant isolates, 83 (83%) harbored *ermA*, 17 (17%) harbored *ermC*, 10 (10%) harbored *ermB*, 4 (4%) harbored *linA*, and 2 (2%) harbored *linB*; 4 (4%) isolates tested negative for these genes. Among gentamicin-resistant MRSA, which represented the aminoglycosides class, out of 62 isolates, 60 (96.8%) harbored *aac(6')/aph(2''),* 52 (83.9%) harbored *aph(3’)-IIIa*, and 6 (9.7%) harbored *ant(4’)-Ia* genes. In the tetracyclines class, of the 31 tetracycline-resistant MRSA isolates, 2 (6.5%) harbored *tetK* and 29 (93.5%) harbored *tetM* genes. Furthermore, in the folate inhibitors class, of the 30 trimethoprim/sulfamethoxazole-resistant MRSA isolates, 4 (13.3%) harbored *dfrA* and 30 (100%) harbored *dfrG* genes*.*

Among macrolide-resistant MRSA, the most common gene combination was *msrA* + *msrB* (27.3%), followed by *ermA* + *msrA* + *msrB* (5%), and *ermA* + *ermB* + *msrA* + *msrB* (2.5%). In the aminoglycosides class, the gene combination *aac(6')/aph(2'')* + *aph(3’)-IIIa* (82.2%) was more common, followed by *aac(6')/aph(2'')* + *ant(4’)-Ia* (4.8%), and *aac(6')/aph(2'')* + *aph(3’)-IIIa* + *ant(4’)-Ia* (1.6%). Finally, among the fluoroquinolones, lincosamides, and folate inhibitors, the *gyrA* + *grlA* (80.7%), *ermA + ermB* (7.0%), and *dfrA* + *dfrG* (13.3%) combinations, respectively, were observed more frequently. The pattern of antimicrobial-resistance gene distribution among resistant MRSA is outlined in Table 2.

**TABLE 2:** Distribution of antimicrobial resistance genes.

Antimicrobial classes	Number of resistant MRSA isolates (%)	Resistance genes (%)
Macrolides	Erythromycin 161 (74.2)	*ermA* 64 (39.7)
		*ermC* 14 (8.7)
		*msrA* 10 (6.2)
		*msrB* 2 (1.2)
		*ermA* + *ermB* 2 (1.2)
		*ermA* + *ermC* 1 (0.6)
		*ermA* + *msrA* 2 (1.2)
		*msrA* + *msrB* 44 (27.3)
		*ermA* + *ermB* + *emrC* 2 (1.2)
		*ermA* + *ermB* + *msrA* 1 (0.6)
		*ermA* + *msrA* + *msrB* 8 (5.0)
		*emrC* + *msrA* + *msrB* 2 (1.2)
		*ermA* + *ermB* + *emrC* + *msrA* 1 (0.6)
		*ermA* + *ermB* + *msrA* + *msrB* 4 (2.5)
		*ermA* + *ermB* + *emrC* + *msrA* + *msrB* 1 (0.6)
		Unknown 3 (1.9)
Lincosamides	Clindamycin 100 (46.1)	*ermA* 68 (68.0)
		*ermC* 13 (13.0)
		*ermA* + *ermB* 7 (7.0)
		*ermA* + *ermC* 1 (1.0)
		*ermA* + *ermB* + *ermC* 3 (3.0)
		*ermA* + *linA* 2 (2.0)
		*ermA* + *linA* + *linB* 2 (2.0)
		Unknown 4 (4.0)
Aminoglycosides	Gentamycin 62 (28.6)	*ant(4’)-Ia* 2 (3.2)
		*aac(6')/aph(2'')* 5 (8.1)
		*aac(6')/aph(2'')* + *ant(4’)-Ia* 3 (4.8)
		*aac(6')/aph(2'')* + *aph(3’)-IIIa* 51 (82.2)
		*aac(6')/aph(2'')* + *aph(3’)-IIIa* + *ant(4’)-Ia* 1 (1.6)
Folate inhibitors	Trimethoprim-sulfamethoxazole 30 (13.8)	*dfrG* 26 (86.7)
		*dfrA* + *dfrG* 4 (13.3)
Tetracyclines	Tetracycline 31 (14.3)	*tetK* 2 (6.5)
		*tetM* 29 (93.5)

***aac(6’)/aph(2’’):*** gene that encodes aminoglycoside-6’-N-acetyltransferase/2’’-O-phosphoryltransferase; ***ant(4’)-Ia*:** gene that encodes aminoglycoside-4’-O-phosphoryltransferase I;***aph(3’)-IIIa:*** gene that encodes aminoglycoside-3’-O-phosphoryltransferase III;***dfrA:*** gene that encodes dihydrofolate reductase A; ***dfrG:*** gene that encodes dihydrofolate reductase B; ***ermA:*** gene that encodes erythromycin ribosomal methylase A; ***ermB:*** gene that encodes erythromycin ribosomal methylase B; ***ermC:*** gene that encodes erythromycin ribosomal methylase C; ***linA:*** gene that encodes lincosamide nucleotidyltransferases A; ***linB:*** gene that encodes lincosamide nucleotidyltransferases B; ***msrA:*** gene that encodes macrolides streptogramins resistance A; ***msrB:*** gene that encodes macrolides streptogramins resistance B; ***tetK:*** tetracycline resistance protein K; ***tetM:*** tetracycline resistance protein M.

### SCC*mec* typing 

The SCC*mec* type IV (57.1%) was the most frequent SCC*mec* type among the MRSA isolates, followed by type III (17.1%), type I (13.4%), type II (9.2%), and type V (1.4%). Four MRSA isolates were nontypable. Isolates of the MRSA SCC*mec* types VI, VII, IX, and X were not detected. The antimicrobial resistance distribution pattern with respect to the MRSA SCC*mec* types is presented in Table 3. 

**TABLE 3:** Antimicrobial resistance distribution between the methicillin-resistant *Staphylococcus aureus* SCC*mec* types.

Antimicrobials	**SCC*mec* types**					
	I (n = 29)	II (n = 20)	III (n = 37)	IV (n = 124)	V (n = 3)	NT (n = 4)
Erythromycin	28 (96.6%) ^c,d^	20 (100%) ^c,d^	33 (89.2%) ^a,c^	75 (60.5%) ^b^	1 (33.3%)	4 (100%)
Ciprofloxacin	28 (96.6%) ^c,d^	20 (100%) ^c,d^	33 (89.2%) ^a,c^	55 (44.0%) ^b^	-	4 (100%)
Clindamycin	28 (96.6%) ^c,d^	19 (95.0%) ^c,d^	32 (86.5%) ^a,c^	18 (14.5%) ^b^	1 (33.3%)	2 (50.0%)
Gentamycin	24 (82.8%) ^e^	2 (10.0%)	29 (78.4%) ^a,e^	3 (2.4%) ^b^	-	4 (100%)
Tetracycline	-	2 (10.0%)	26 (70.3%) ^a,f^	2 (1.6%) ^b^	1 (33.3%)	-
Trimethoprim-sulfamethoxazole	-	1 (5.3%)	20 (54.1%) ^a,f^	5 (4.0%) ^b^	-	4 (100%)

NT: non-typable. Data are indicated by the number of isolates (%).

a MRSA SCC*mec* type III was more multidrug-resistant (p < 0.001).

b MRSA SCC*mec* type IV was more multidrug-susceptible (p < 0.001).

c MRSA SCC*mec* types I, II, and III were more resistant to erythromycin, ciprofloxacin, and clindamycin, than MRSA SCC*mec* type IV (p < 0.001).

d MRSA SCC*mec* types I and II were more resistant to erythromycin, ciprofloxacin, and clindamycin, than MRSA SCC*mec* type V (p < 0.001).

e MRSA SCC*mec* types I and III were more resistant to gentamycin than MRSA SCC*mec* types IV and V (p < 0.001).

f MRSA SCC*mec* type III was more resistant to trimethoprim-sulfamethoxazole and tetracycline than most MRSA SCC*mec* types I, II, and IV (p < 0.001)

In general, the MRSA SCC*mec* type III strains exhibited higher multidrug resistance (p < 0.001). In contrast, the MRSA SCC*mec* type IV strains were more multidrug-susceptible compared to the other SCC*mec* types (p < 0.001). The MRSA SCC*mec* type I, type II, and type III strains were more resistant to ciprofloxacin, clindamycin, and erythromycin than the MRSA SCC*mec* type IV strains, which were significantly susceptible to the same antimicrobials (p < 0.001). Similarly, the MRSA SCC*mec* type I and type II strains were more resistant to these antimicrobials than MRSA SCC*mec* type V strains, which were significantly susceptible (p < 0.001). In addition, the MRSA SCC*mec* type I and type III strains were more resistant to gentamycin than the MRSA SCC*mec* type IV and type V strains, that were susceptible to the same antimicrobial (p < 0.001). Lastly, the MRSA SCC*mec* type III strains were more resistant to trimethoprim-sulfamethoxazole and tetracycline than most MRSA SCC*mec* type I, type II, and type IV strains, which were susceptible to the same antimicrobials (p < 0.001).

## DISCUSSION

In the last two decades, the proportion of MRSA has increased worldwide^18^. At present, MRSA may be considered the first class of multidrug-resistant (MDR) pathogens, based on the emergence of the concomitant resistance of MRSA to multiple commonly used non-beta-lactam antimicrobials (for e.g., aminoglycosides, macrolides, fluoroquinolones, and tetracycline)^25^ ^-^ ^27^.

In the present study, 177 MRSA (81.6%) isolates exhibited resistance to at least one of the non-beta-lactam antimicrobials tested, which is indicative of the high resistance rates for erythromycin, ciprofloxacin, and clindamycin antimicrobials. These resistance rates are in accordance with findings from other studies in southern^28^ ^-^ ^30^and other regions of Brazil^31^ ^,^ ^32^. 

Erythromycin and clindamycin are members of the macrolide-lincosamide-streptogramin B (MLS_B_) family, which exhibit excellent potential in MRSA infections and are frequently used to treat staphylococcal skin and soft tissue infections (SSTIs)^12^ ^,^ ^13^ ^,^ ^33^. *erm* gene-mediated resistance to MLS_B_ can be expressed in constitutive (cMLS_B_ phenotype) or inducible (iMLS_B_ phenotype) forms^18^ ^,^ ^34^ ^,^ ^35^. In this study, the prevalence of cMLS_B_ was 38.7%, whereas other studies conducted in Brazil reported cMLS_B_ resistance of approximately 14.3% and 68.2%^34^ ^,^ ^36^. 

Besides, an important issue in the application of clindamycin is the inducible resistance owing to the presence of methylase synthesis inducers, such as erythromycin, which leads to increased failure in clinical therapeutic applications^18^ ^,^ ^34^. In this study, a prevalence of the iMLS_B_ phenotype was observed among 7.4% of the MRSA isolates tested, which is consistent with that reported by Bottega et al (7.9%)^36^, and higher than that reported by Pereira et al. (4.5%)^34^, with both studies conducted in Brazil.

The distribution of resistance genes detected in this analysis demonstrates that *ermA* (39.6%) was the predominant gene compared to *ermC* (9.7%) and *ermB* (5.1%). In contrast, in another Brazilian study, it was shown that *ermC* (38.6%; 17/44) was identified more frequently than *ermA* (9.1%; 4/44)^9^ ^,^ ^37^ ^-^ ^41^. In this study, one MRSA isolate carried both *ermA* and *ermC*, which encode proteins for erythromycin and clindamycin resistance. The coexistence of these genes in MRSA isolates was also observed in other studies^9^ ^,^ ^34^ ^,^ ^42^. 

Among the *msr* genes, *msrA*, which confers resistance to macrolides and type B streptogramins, had the highest prevalence (33.6%), followed by *msrB* (28.1%). In contrast to the data from this study, resistance via efflux pumps (associated with *msrA*/*msrB*) was not detected in MRSA in a study by Khodabandeh et al.^9^, whereas Sarrou et al.^42^detected only *msrA* in MRSA isolates. The *msrA* + *msrB* gene combination of resistance was more prevalent in this study. Additionally, the *ermA* + *ermB* + *msrA* + *msrB* gene combination was detected in four isolates. These findings are consistent with those of a study on the development of genotype prevalence in Serbia performed by Misic et al.^43^, in which similar results of genetic combinations were reported. 

The predominance of MRSA in SSTIs and their treatment using ciprofloxacin consequently led to an increase in fluoroquinolone resistance, and thereby limited the therapeutic use of this class of antimicrobials^44^ ^,^ ^45^. In this study, 64.5% of MRSA isolates were resistant to ciprofloxacin. In two studies with MRSA isolated from seven hospitals in Rio de Janeiro, Brazil, resistance to fluoroquinolones varied between 60.6% and 93%^32^ ^,^ ^46^while in another study that used isolates collected from three cities in a southern Brazilian state, 79% of the MRSA isolates exhibited fluoroquinolone resistance^30^. 

In this study, the rates for trimethoprim-sulfamethoxazole, tetracycline, and gentamicin resistance were observed to be low, which was consistent with recent reports from other studies conducted in Brazil^29^ ^,^ ^31^. 

Trimethoprim-sulfamethoxazole (folate inhibitors class) is an alternative choice for the treatment of mild to moderate SSTIs caused by MRSA, based on the results of susceptibility tests^4^ ^,^ ^11^. The presence of the *dfrG* gene was confirmed in 86.7% of MRSA isolates, and the association between *dfrA* and *dfrG* was confirmed in 13.3%. Moreover, Coelho et al.^47^ compared *S. aureus* isolates collected from Portuguese-speaking African countries with a Brazilian MRSA clone (ST239-III), and observed 78% prevalence of the *dfrG* gene, 19% of the *dfrA* gene, or 3% of both.

Aminoglycosides constitute an important class of antimicrobials, especially for the treatment of complicated staphylococcal infections synergistically with glycopeptides or beta-lactams^48^. In an attempt to confirm the resistance to aminoglycosides in MRSA, the presence of genetic elements that encode AMEs was evaluated, with 82.2% of MRSA exhibiting the *aac(6')/aph(2'')*/*aph(3’)-IIIa* gene association. Previous reports showed the presence of this association among 9% and 55.5% isolates^10^ ^,^ ^49^. Tetracycline has exhibited clinical efficacy in cases of community-associated MRSA SSTIs^50^. In this study, the presence of *tetK* and *tetM* genes was observed in 6.5% and 93.5% of isolates, respectively.

In contrast, all the MRSA isolates tested were susceptible to linezolid and vancomycin, as observed in other Brazilian studies^51^ ^-^ ^54^. Currently, resistance to oxazolidinones (including linezolid) among *S. aureus* is rare, whereas prolonged exposure to vancomycin leads to the emergence of MRSA with reduced vancomycin susceptibility, and the strains are categorized as vancomycin-intermediate *S. aureus* (VISA) and heterogeneous VISA (hVISA)^55^, as reported in other studies conducted in Brazil^28^ ^-^ ^56^. Nevertheless, vancomycin remains the first-line therapeutic choice for the treatment of invasive MRSA infections, such as bacteremia, pneumonia, and osteoarticular infection; linezolid is an alternative for the treatment of invasive hVISA and VISA infections^3^ ^,^ ^4^.

SCC*mec* typing provides useful information regarding resistance to antimicrobials and the origin of *S. aureus* strains^57^. In our study, SCC*mec* IV and III were the most common SCC*mec* types, which is consistent with findings reported earlier^57^ ^-^ ^59^. In addition, the MRSA SCC*mec* type III strains exhibited higher multidrug resistance, whereas the MRSA SCC*mec* type IV were more multidrug-susceptible compared to other SCC*mec* types. Previous studies have shown that HA-MRSA isolates generally contain SCC*mec* types I, II, or III, which confer resistance to non-beta-lactam antimicrobials and tend to lead to multidrug-resistance^7^ ^-^ ^60^. Furthermore, SCC*mec* type IV was most commonly detected among the MRSA isolates, and this characteristic is often observed in CA-MRSA strains, which are generally susceptible to non-beta-lactam antimicrobials and harbor SCC*mec* types IV or V.

Our study has certain limitations. First, we were unable to test other therapeutic options, such as ceftaroline, daptomycin, and tigecycline. Second, we could not determine the MICs for all the antimicrobials tested. Despite these limitations, the HA-MRSA isolates in our setting were confirmed to be MDR, which limits the therapeutic options available for the treatment of infections caused by such MRSA isolates. Third, the isolates included in this study were not genotyped to assess the clonality. In this study, high erythromycin, ciprofloxacin, and clindamycin resistance rates were observed, and the isolates exhibited considerable diversity of genes related to non-beta-lactam resistance mechanisms in MRSA strains. This indicates the urgency for the development of alternative therapeutic options. Despite the fact that multidrug resistance is increasing in the study setting, linezolid and vancomycin appear to be effective therapeutic options for MDR-MRSA strains. The study data provide information regarding the resistance profile of MRSA isolates from South Brazil, and along with data on the clinical conditions of the patients, it can contribute to the clinical decision-making process.
